# Electroacupuncture Modulates 5-HT_4R_-Mediated cAMP/PKA Signaling to Improve Intestinal Motility Disorders in a Thy1-*α*Syn Parkinson's Mouse Model

**DOI:** 10.1155/2022/8659462

**Published:** 2022-10-28

**Authors:** Lin Shen, Zhaoqin Wang, Rude Huang, Luyi Wu, Yan Huang, Qin Qi, Rui Zhong, Yiyi Chen, Lingjie Li, Huangan Wu

**Affiliations:** ^1^Shanghai Research Institute of Acupuncture and Meridian, Shanghai University of Traditional Chinese Medicine, Shanghai 200030, China; ^2^Community Health Service Center of Malu Town, Jiading District, Shanghai 201801, China; ^3^Department of Aeronautics and Astronautics, Shanghai Key Laboratory of Acupuncture Mechanism and Acupoint Function, Fudan University, Shanghai 200433, China; ^4^Key Laboratory of Acupuncture-Moxibustion and Immunological Effects, Shanghai University of Traditional Chinese Medicine, Shanghai 200030, China; ^5^Shanghai University of Traditional Chinese Medicine, Shanghai 201203, China; ^6^Yueyang Hospital of Integrated Traditional Chinese and Western Medicine, Shanghai University of Traditional Chinese Medicine, Shanghai, China

## Abstract

Constipation is one of the most common nonmotor symptoms in patients with Parkinson's disease (PD) and often occurs before motor symptoms. Electroacupuncture effectively improves the symptoms of constipation in patients with PD. In the present study, we used thymus cell antigen 1-*α*-synuclein (Thy1-*α*Syn) transgenic mice as a model of intestinal motility disorders in PD to determine the therapeutic effect of electroacupuncture and the underlying mechanisms. Electroacupuncture significantly improved fecal excretion and accelerated the rate of small-intestinal propulsion in Thy1-*α*Syn mice by upregulating the serotonin concentration and the expression of the serotonin 4 receptor. Consequently, the downstream cyclic AMP/protein kinase *A* (cAMP/PKA) pathway was affected, and to upregulate and downregulate, the expression of substance *P* was upregulated, and the expression of calcitonin gene-related peptide was downregulated. In summary, electroacupuncture improved intestinal motility in PD mice by affecting serotonin levels, serotonin 4 receptor expression, and the cAMP/PKA pathway, providing a potentially effective and promising complementary and alternative therapy for relieving constipation symptoms in patients with PD.

## 1. Introduction

Parkinson's disease (PD) is a progressive neurodegenerative disease [1]. The prevalence of PD has increased recently, with more than 1% of the population of >60 years and more than 3% of the population of >80 years suffering from this disease in western industrialized countries [2]. In China, the prevalence of PD is similar to that in western countries, with a prevalence of 1.7% in the population of ≥65 years, and the number of patients with PD is expected to increase substantially as the population ages [3]. The main symptoms of PD include nonmotor symptoms (NMSs) and motor symptoms [4, 5]. Patients with PD often present with constipation [6], which is the most common NMS [7], with an incidence of approximately 24%–70% [8]. Constipation in PD is primarily slow-transit constipation, which is characterized by intestinal motility disorders, with the clinical manifestations of infrequent bowel movement and dry and hard stool [9]. Constipation seriously affect the quality of life of patients with PD [10].

The pathogeneses of PD and constipation in PD have not been fully elucidated. Existing studies suggest that abnormal accumulation of alpha-synuclein (*α*Syn) in the brain is closely related to the pathogenesis of PD [11] and is an important target for PD treatment [12]. The pathogenesis of constipation in PD may be related to the degeneration of the enteric nervous system and the imbalance of intestinal microbiota involved in the brain-gut axis [13]; the enteric nervous system plays a particularly important role [14]. A previous study showed abnormal aggregation of *α*Syn in the colonic nerves of patients with early-stage PD [15]. Braak et al. proposed that the abnormal accumulation of *α*Syn is likely initiated in the enteric nervous system and proposed the theory of the brain-gut-microbiome axis, arguing that the pathogenesis of PD originates from the gut. Another study showed that in patients with PD, the intestinal microbiota is disturbed, and the intestinal mucosal barrier is disrupted after microbial metabolites stimulate the intestinal mucosa. *α*Syn is abnormally accumulated in the intestine and transferred to the brain along the vagus nerve, leading to the onset of PD, eventually causing motor symptoms, NMSs, and constipation [16]. At present, drug therapy is the main treatment for constipation in patients with PD, which, although effective, is also associated with adverse reactions, such as diarrhea, dry mouth, and headache [17, 18]. Considering the high incidence of constipation in patients with PD, an effective treatment with minimal adverse effects is necessary.

The potential therapeutic effects of traditional medicines, natural plant extracts, and traditional remedies have gained attention in recent years [19–24]. Some traditional medicines and therapies have been found to have neuroprotective effects [25–28] and have been widely used in the treatment of neurodegenerative diseases or other related diseases [29–33]. Acupuncture is a physical therapy of traditional Chinese medicine, which has been used in China for more than 3000 years. Recent studies have demonstrated the neuroprotective role of acupuncture in the treatment of PD [34, 35]. Electroacupuncture is implemented by connecting the acupuncture needles to a low-frequency electroacupuncture instrument to stimulate the meridians and acupoints using microcurrents. Electroacupuncture can improve gastrointestinal dysfunction by modulating the enteric nervous system [36]. In recent years, the efficacy of acupuncture and electroacupuncture for treating functional constipation has reached an international consensus [37–39]. Previous studies have shown that electroacupuncture is superior in improving constipation symptoms in patients with PD, especially in improving anorectal dynamics [40–42]. Although electroacupuncture can relieve constipation in patients with PD, its underlying mechanism is still unclear, which forms the basis of our research.

Abnormal intestinal motility, decreased 5-hydroxytryptamine (5-HT) levels, and downregulation of the serotonin 4 receptor (5-HT_4R_) in the gastrointestinal tract are associated with the pathogenesis of PD. Shi reported decreased 5-HT_4R_ expression in the hypothalamus and hippocampus of 1-methyl-4-phenyl-1, 2, 3, 6-tetrahydropyridine-induced PD mice, whereas injection of the 5-HT_4R_ antagonist GR125487 aggravated the neurodegenerative processes in the mouse nigrostriatal pathway and modulated the inflammation-related intestinal microbiota composition [43]. More importantly, abnormal intestinal motility is closely related to gastrointestinal 5-HT_4R_ [44], which is an important target for treating constipation in PD. When the gut is stimulated, 5-HT is released from the gut to excite 5-HT_4R_, affecting the downstream cyclic AMP/protein kinase *A* (cAMP/PKA) pathway, resulting in the secretion and release of substance *P* (SP), calcitonin gene-related peptide (CGRP), and other neurotransmitters, which cause the coordinated contraction and relaxation of gastrointestinal smooth muscle. In clinical practice, mosapride is a commonly used 5-HT_4R_ agonist for treating constipation [45]. A previous study demonstrated that electroacupuncture effectively improves the symptoms of constipation in patients with irritable bowel syndrome and regulates the abnormal 5-HT_4R_ expression in the colon of these patients [46]. This result was also verified in an animal model in our previous study [47]. Therefore, we hypothesized that the regulation of 5-HT_4R_ and the downstream cAMP/PKA pathway may be a potential target of electroacupuncture for treating constipation in PD.

In the present study, we used a transgenic mouse model overexpressing *αSyn* under the control of the thymus cell antigen 1 (*Thy1*) promoter to explore the mechanisms underlying the beneficial effects of electroacupuncture on intestinal motility disorders in PD.

## 2. Materials and Methods

### 2.1. Experimental Animals

Thy1-*α*Syn transgenic mice overexpressing *αSyn* under the control of the *Thy1* promoter in a C57BL/6J background were purchased from Jackson Laboratory (Bar Harbor, ME). The mice were then bred by Jiangsu Jicui Yaokang Biotechnology (Jiangsu Province, China, license number SCXK (Jiangsu) 2018–0008). The transgenic mice were mated with C57BL/6J mice, and their offspring were used for experiments; wild-type littermates of the transgenic offspring served as controls. During the experiments, all animals (22–24 weeks old) were housed in the specific pathogen-free animal facility of Yueyang Hospital affiliated with Shanghai University of Traditional Chinese Medicine, with a 12/12 h light-dark cycle, a room temperature of 20°C ± 2°C, and a relative humidity of 50%–70%. All animals were acclimated for a week before the experiments, which were approved by the Animal Ethics Committee of Yueyang Hospital affiliated with Shanghai University of Traditional Chinese Medicine (approval number YYLAC-2020-070). All animal experiments were conducted in accordance with the *Guides on the Humane Treatment of Laboratory Animals* [48].

### 2.2. Drugs and Reagents

Activated carbon powder and gum arabic were obtained from Shanghai Sangon Biological Engineering Technology and Service; mosapride citrate dispersible tablets were obtained from Chengdu Kanghong Pharmaceutical Group (Sichuan Province, China); the PrimeScript™ RT Reagent kit with gDNA Eraser (Perfect Real Time, RR047A) and TB Green® Premix Ex Taq™ (Tli RNase H Plus) were obtained from Takara; the SABC-HRP Kit (P0612) was obtained from Beyotime Biotechnology (Jiangsu Province, China); anti-5-HT_4R_ antibody (1 : 200 for immunohistochemistry (IHC) and 1 : 1000 for western blot (WB), ab60359) was obtained from Abcam; anti-PKA antibody (1 : 1000, AF7746) and anti-SP antibody (1 : 2000, DF7522) were obtained from Affinity; anti-CGRP antibody (1 : 1000, ab189786) was obtained from Abcam; and the 5-HT enzyme-linked immunosorbent assay (ELISA) kit (SDM0131) and the cAMP ELISA kit (SDM0130) were obtained from Simuwu Biotechnology (Shanghai, China).

### 2.3. Experimental Grouping

After a week of acclimation, 18 Thy1-*α*Syn transgenic mice were randomly divided into the following three groups: the PD model group (*n* = 6), the PD model with electroacupuncture treatment group (*n* = 6), and the PD model with Western medicine treatment group (*n* = 6). Six wild-type mice were included as the normal control group. The male-to-female ratio of animals was 1 : 1.

### 2.4. Interventions

The intervention in the electroacupuncture group was electroacupuncture at *Tianshu* (ST25, bilateral) acupoints and regular acupuncture at the *Baihui* (DU20) acupoint using sterile Huatuo acupuncture needles. For electroacupuncture, the needles were connected to an acupoint nerve stimulator (HANS) with a current intensity of 2 mA and a dilatational wave frequency alternating between 2 and 15 Hz; the treatment duration was 20 min [49, 50]. This intervention was performed once daily for seven consecutive days. Electroacupuncture of the bilateral *Tianshu* acupoints was performed using bi-electrode needles as described previously [51]. Specifically, two needles (0.18 mm in diameter and 13 mm in length) were wrapped with medical tape as an insulating layer to only expose the handle ends and needle ends. The handle ends of the needles were then connected to the anode and cathode of the electroacupuncture device. The *Tianshu* acupoints were located approximately 20 mm above the pubic symphysis and approximately 5 mm away from the midline of the abdomen on each side. At these acupoints, a straight puncture was performed to a depth of 3–5 mm. Regular acupuncture of the *Baihui* (DU20) acupoint, located at the midpoint of the anterior midline and the line connecting the two ear tips, at the middle of the parietal bone, was performed using a needle of 0.18 mm in diameter and 13 mm in length via a posterior-inclined puncture to a depth of 2 mm, with rotating but no lifting of the needle. For the acupoint positions, we referred to the *Experimental Acupuncture and Moxibustion* (first edition, 2009) published by People's Medical Publishing House (Beijing, China) [52].

In the Western medicine group, mosapride suspension was prepared by grinding mosapride citrate dispersible tablets (5 mg/tablet) and dissolving them in double-distilled water. The suspension was shaken before each use. With reference to the *Methods of Pharmacological Experiments* [53], on the basis of the body surface area of a 60-kg adult human and the clinical dose (5 mg, thrice daily), the daily dose of mosapride for each mouse was 0.0017 mg/g, once a day for 7 days, administered by gavage.

All animals were handled in the same way. The animals in the electroacupuncture group, normal control group, and PD model group were given normal saline by gavage (the same gavage volume as in the western medicine group).

### 2.5. General Observation of the Mice

During the experimental period, the general conditions of the mice, including defecation, limb tremor, and shaking, were monitored.

### 2.6. Fecal Output Test

A day before sampling, measurement of the fecal output was performed by placing each mouse into a transparent box (20 cm × 35 cm × 15 cm) and counting the fecal pellets every 5 min for a total of 15 min [54].

### 2.7. Rotarod Test

A rotarod apparatus purchased from Chengdu TME Technology was used to measure the motor performance of mice in each group and to evaluate the motor impairments of the PD mouse model. The overall rotarod performance (ORP) was recorded as described by Rozas et al. [55]. Before the experiments, all mice were subjected to accelerate rotarod training on the apparatus, and mice that stayed on the rotating rod for 120 s at a speed of 20 rpm were selected for the actual rotarod test. On day 7 of intervention, all mice underwent accelerated rotarod training again. In the rotarod test, the durations that mice stayed on the rotating rod at rotation speeds of 10, 15, 20, 25, and 30 rpm were recorded, with a maximum residence time of 300 s at each rotation speed. The ORP score of each mouse was calculated using the trapezoidal method [56, 57].

### 2.8. Assessment of the Small-Intestine Propulsion

Small-intestine propulsion was assessed to evaluate the intestinal function of mice to determine the presence of intestinal motility disorders. After completion of the fecal excretion test, all mice were fasted for 24 h, and each mouse was given an activated carbon suspension at a dose of 0.1 mL/10 g (gum arabic and activated carbon powder at a ratio of 2 : 1 were dispersed in water) by gavage. All animals were sacrificed 20 min later to collect the intestinal tract from the pylorus to the ileocecal region. The entire length of the small intestine and the furthest distance advanced by the charcoal suspension in the small intestine (i.e., the distance from the pylorus to the front of the charcoal suspension) were measured to calculate the small-intestine propulsion (*P*, %) according to the following equation: *P* (%) = transit distance of carbon suspension (cm)/length of the entire small-intestine (cm) × 100% [45].

### 2.9. Specimen Collection and Processing

A colon segment of approximately 8 cm in length starting from approximately 2 cm above the anus was collected. The colon segment was then washed with normal saline and cut into two segments, one was stored at −80°C and the other was fixed in 4% paraformaldehyde.

### 2.10. Immunohistochemistry

The protein expression of 5-HT_4R_ in the mouse colon tissue was detected by IHC. In brief, after conventional deparaffinization, hydration, antigen retrieval, and blocking, the colon tissue sections were incubated with anti-5-HT_4R_ primary antibody (1 : 200) at 4°C overnight and subsequently analyzed using a SABC-HRP (P0612, Beyotime) detection kit. After being washed with phosphate-buffered saline, the tissue sections were subjected to color development using a 3, 3′-diaminobenzidine colorimetric substrate, nuclear counterstaining by hematoxylin solution, and conventional dehydration and mounting. The cells with brown cytoplasmic granular staining were considered 5-HT_4R_-positive. The cells were quantified by randomly selecting three fields of view under light microscopy, and the positive staining area (Area) and integrated optical density (IOD) of each image were calculated using Image-Pro Plus software to obtain the average positive staining area (AOD = IOD/Area) of each colon section.

### 2.11. Quantitative Reverse Transcription PCR

To measure the mRNA expression levels of 5-HT_4R_, PKA, CGRP, and SP in the mouse colon tissue of different groups, frozen colon tissue samples were ground, pulverized, homogenized, and centrifuged. Total RNA was isolated from the supernatant using TRIzol Reagent and reverse transcribed to prepare the cDNA templates for quantitative reverse transcription PCR (RT-qPCR). Glyceraldehyde-3-phosphate dehydrogenase (*GAPDH*) was used as an internal reference gene for calculating the relative mRNA expression of 5-HT_4R_, PKA, CGRP, and SP. The total volume used for the RT-qPCR reactions was 20 *μ*L. The PCR tubes were placed in a fluorescence qPCR cycler for amplification. The relative mRNA expression of individual target genes was determined using the 2^−ΔΔCt^ method.  Table 1 shows the mouse-specific primer sequences used for RT-qPCR in this study.

### 2.12. Western Blot

Frozen colon tissue was homogenized in RIPA buffer, samples were centrifuged, and the supernatant was collected. The quantification of total protein was performed using a bicinchoninic acid assay. After denaturation and adjustment of the concentration, the protein samples were separated by electrophoresis and transferred onto protein membranes. Subsequently, the membranes were blocked in 5% skim milk solution for 1 h at room temperature, followed by overnight incubation with primary antibodies (anti-5-HT_4R_, 1 : 1000, ab60359, Abcam; anti-PKA, 1 : 1000, AF7746, Affinity; anti-SP, 1 : 2000, DF7522, Affinity; or anti-CGRP, 1 : 1000, ab189786, Abcam) on a shaker at 4°C. After being washed, the membranes were further incubated with secondary antibodies at room temperature for 1 h. Finally, the membranes were washed, and protein bands were visualized and imaged using a Bio-Rad imaging system. The gray values of individual protein bands were quantified using ImageJ software.

### 2.13. ELISA

The 5-HT and cAMP concentrations in the colon tissue samples were measured using 5-HT and cAMP ELISA kits (5-HT, SDM0131, Simuwu Biotechnology; cAMP, SDM0130, Simuwu Biotechnology) with standard curves following the manufacturer's instructions.

### 2.14. Statistical Analysis

The SPSS 21.0 software package (IBM, Armonk, NY) was used for statistical analysis. Normality and homogeneity of the variance were tested. Normally distributed data are presented as the mean ± standard deviation ($\bar{x\;}$ ± *s*). The data were compared between the two groups using a two-sample*t*-test (model identification) and between multiple groups using one-way analysis of variance (pairwise comparison using Fisher's least significant difference method (homogeneous variance) or Dunnett's T3 method (unequal variance)). The nonnormally distributed data are presented as median and quartiles (median (*P*_25_, *P*_75_)) and were compared using the Kruskal–Wallis H rank-sum test for differences between multiple groups and Dunnett's T3 method for pairwise comparison. *P* values of <0.05 were considered statistically significant.

## 3. Results

### 3.1. Observation of General Conditions

The generation conditions of mice in each group, including general appearance, hair, drinking, and eating performance, behavior, and defecation, were monitored (Table 2). Regarding the motor symptoms, the mice in the electroacupuncture and Western medicine groups still had involuntary tremor and limb shaking, similar to the mice in the PD model group. Defecation of the mice in the PD model group was significantly less than that of the normal control group. Electroacupuncture and Western medicine interventions increased defecation in PD mice.

### 3.2. Electroacupuncture Improved Fecal Excretion and Increased Small-Intestine Propulsion of Thy1-*α*Syn Transgenic Mice

**Figure 1:**
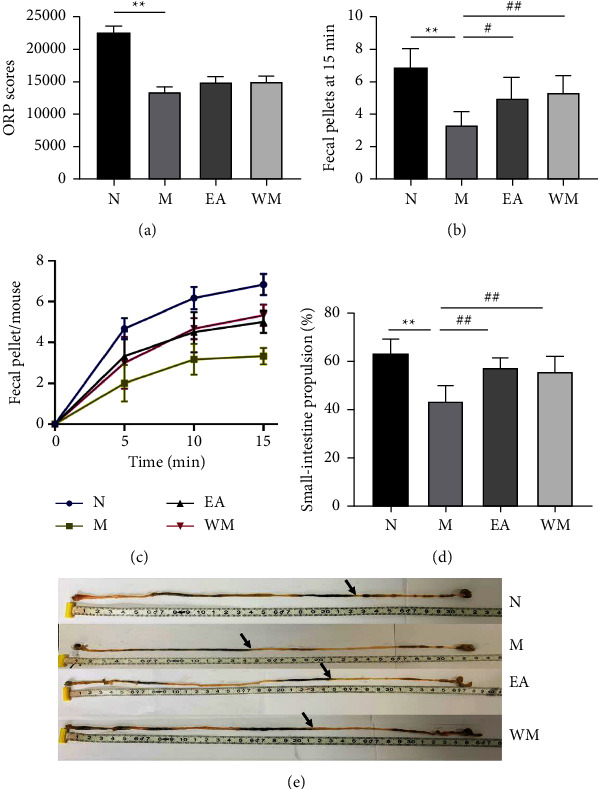
Thy1-*α*Syn transgenic mice had motor impairment and intestinal motility disorder, and electroacupuncture significantly improved the fecal output and small-intestinal propulsion. (a) Overall rotarod performance (ORP) scores. (b) Total fecal pellets produced in 15 min. (c) Fecal output at different time intervals within the detection period. (d, e) Small-intestine propulsion and representative images showing the dissected intestines of mice in the normal control (N), PD model (M), electroacupuncture (EA), and western medicine (WM) groups. ^∗^*P* < 0.05 and ^∗∗^*P* < 0.01 relative to the normal control group; ^#^*P* < 0.05 and ^##^*P* < 0.01 relative to the PD model group.

We further used the rotarod test to evaluate the exercise capacity in different groups of mice and used fecal output in 15 min and small-intestine propulsion to evaluate the severity of intestinal motility disorders of the mice. The results of the rotarod test showed that the ORP of the PD mice was significantly lower than that of the normal control mice (*P* < 0.01), whereas electroacupuncture and Western medicine increased the ORP of the PD mice, although no significant difference was found compared with the PD model group (*P* = 0.05, Figure 1(a)).

Measurement of fecal excretion showed that the fecal output of the PD model group was significantly less than that of the normal control group (*P* < 0.01). The fecal output (within 15 min) of the electroacupuncture and Western medicine groups was significantly higher than that of the PD model group (*P* < 0.05, Figure 1(b)), and the fecal output during the first 5 min interval was higher than that during the second and third 5 min intervals (Figure 1(c)).

The PD mice in the model group were also found to have significantly slower propulsion in the small intestine than the normal control mice (*P* < 0.01), whereas electroacupuncture and Western medicine significantly improved the small-intestine propulsion of the PD mice (*P* < 0.05) compared with the PD mice without any intervention (Figures 1(d) and 1(e)).

### 3.3. Electroacupuncture Upregulated the 5-HT Concentration and 5-HT_4R_ Expression in the Colon Tissue of Thy1-*α*Syn Mice

**Figure 2:**
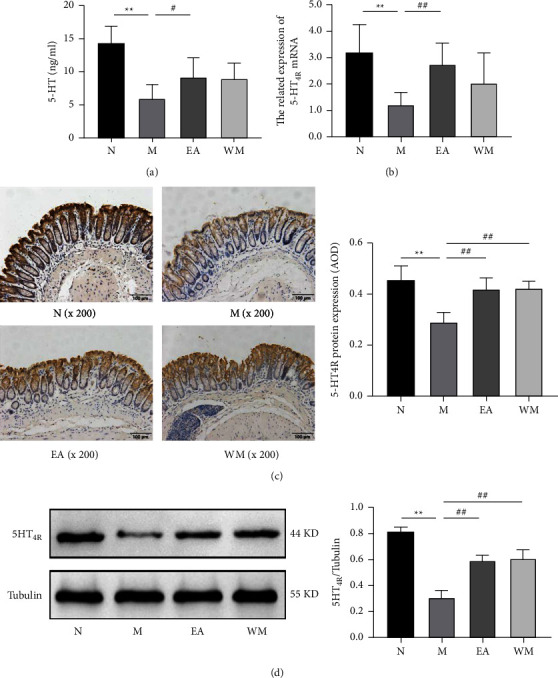
Effects of electroacupuncture on the 5-HT concentration and 5-HT_4R_ expression in the colon tissue of Thy1-*α*Syn transgenic mice. (a) Serotonin (5-HT) concentrations. (b) mRNA expression of 5-HT_4R_. (c) Representative images showing the distribution of 5-HT_4R_ expression. (d) Protein expression of 5-HT_4R_ in the mouse colon tissue of the normal control (N), PD model (M), electroacupuncture (EA), and western medicine (WM) groups. ^∗∗^*P* < 0.01 relative to the normal control group; ^#^*P* < 0.05 and ^##^*P* < 0.01 relative to the PD model group.

The 5-HT concentration in the colon tissue of the PD model mice was significantly lower than that in the normal control group (*P* < 0.01). Electroacupuncture significantly increased the 5-HT concentration in the colon tissue (*P* < 0.05, Figure 2(a)), whereas the effects of Western medicine on the 5-HT concentration in the colon tissue were not significant.

The RT-qPCR, WB, and IHC analyses revealed that the mRNA and protein expression levels of 5-HT_4R_ in the mouse colon tissue of the PD model group were significantly lower than that in the normal control group (*P* < 0.05). The mRNA and protein expression levels in the mouse colon tissue of the electroacupuncture group were significantly elevated compared with the PD model group (*P* < 0.05); similarly, the protein expression in the colon tissue of the Western medicine group was also significantly higher than that of the PD model group (*P* < 0.01, Figures 2(b)–2(d)).

### 3.4. Electroacupuncture Activated cAMP/PKA Signaling in the Colon Tissue

**Figure 3:**
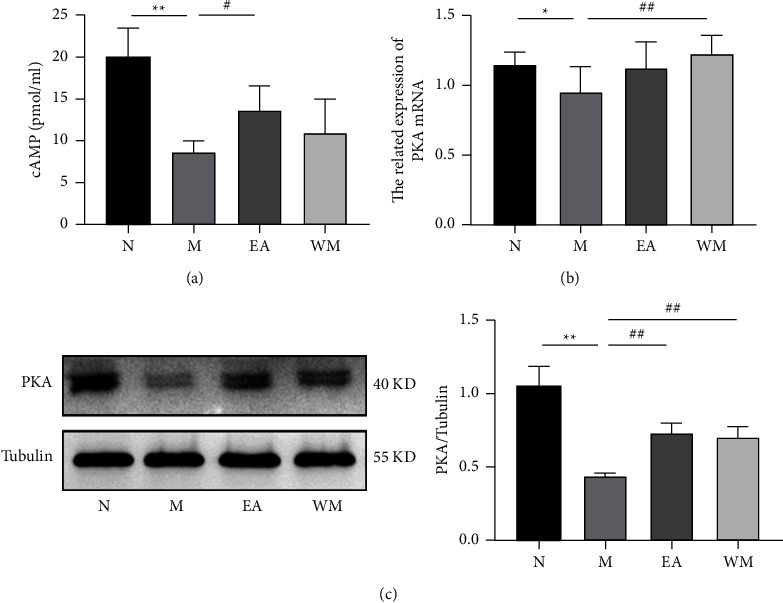
Effects of electroacupuncture on the cAMP/PKA signaling pathway in Thy1-*α*Syn transgenic mice. (a) cAMP concentrations. (b) mRNA expression of PKA. (c) Protein expression of PKA in mouse colon tissue of the normal control (N), PD model (M), electroacupuncture (EA), and western medicine (WM) groups. ^∗^*P* < 0.05 and ^∗∗^*P* < 0.01 relative to the normal control group; ^#^*P* < 0.05 and ^##^*P* < 0.01 relative to the PD model group.

The cAMP concentration and the mRNA and protein expression levels of PKA in the mouse colon tissue of the PD model group were significantly lower compared with the normal control group (*P* < 0.05). Electroacupuncture significantly elevated the cAMP concentration and PKA protein expression levels in the colon tissue of the PD mice compared with the PD model group (*P* < 0.05, *P* < 0.01; Figures 3(a) and 3(c)), and Western medicine also significantly elevated the mRNA and protein expression levels of PKA in the colon tissue of PD mice (*P* < 0.05, Figures 3(b) and 3(c)).

### 3.5. Effects of Electroacupuncture on SP and CGRP Neurotransmitters

**Figure 4:**
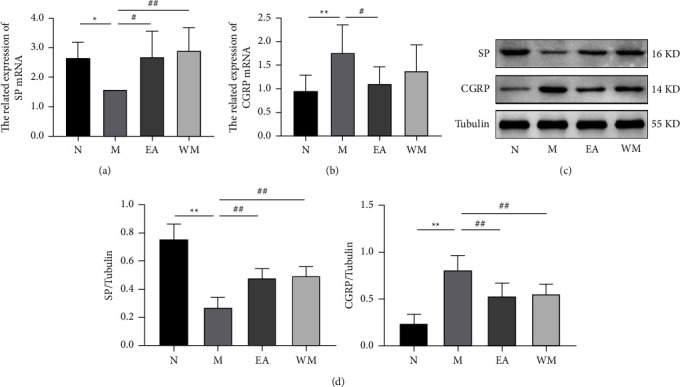
Effects of electroacupuncture on the expression of SP and CGRP neurotransmitters in the colon tissue of Thy1-*α*Syn transgenic mice. (a) mRNA expression of SP. (b) mRNA expression of CGRP. (c) Representative western blot images showing the protein expression levels of SP and CGRP. (d) Protein expression in the mouse colon tissue of the normal control (N), PD model (M), electroacupuncture (EA), and western medicine (WM) groups. ^∗^*P* < 0.05 and ^∗∗^*P* < 0.01 relative to the normal control group; ^#^*P* < 0.05 and ^##^*P* < 0.01 relative to the PD model group.

Our experimental data showed that the protein and mRNA expression levels of SP in the mouse colon tissue of the PD model group were significantly lower than those in the normal control group (*P* < 0.05), whereas the protein and mRNA expression levels of CGRP in the mouse colon tissue of the PD model group were significantly higher (*P* < 0.01). Electroacupuncture and Western medicine significantly increased the protein and mRNA expression levels of SP in the PD mouse colon tissue (*P* < 0.05, Figures 4(a), 4(c), and 4(d)). Electroacupuncture significantly reduced the protein and mRNA expression levels of CGRP in the PD mouse colon tissue (*P* < 0.05), whereas Western medicine significantly reduced the protein expression levels of CGRP in the PD mouse colon tissue (*P* < 0.05, Figures 4(b)–4(d)).

## 4. Discussion

NMSs have gained increasing attention as a result of the recent in-depth clinical and experimental research on PD. Constipation in PD has been recognized as the most prominent early symptom of PD [58], and its occurrence may precede motor symptoms in PD, even years before other PD symptoms occur [59, 60]. Patients with PD with constipation have more severe motor symptoms than patients with PD without constipation. In addition, patients with PD with constipation before motor symptoms have been shown to have a faster progression of motor symptoms [61]. Degeneration of the enteric nervous system is an important factor leading to the pathogenesis of constipation in PD. The enteric nervous system is a part of the autonomic nervous system and regulates intestinal motor functions, including colonic peristalsis. Abnormalities of the enteric nervous system cause colonic smooth muscle dysfunction and persistent inhibition of intestinal contractions, resulting in reduced intestinal motility, delayed intestinal movement, and ultimately constipation [62]. Abnormal accumulation of *α*Syn in the colonic nerve occurs in the early stage of PD. The misfolded *α*Syn in the enteric nervous system leads to the formation of Lewy bodies, disturbance of the enteric nervous system and related transmitters, and reduced intestinal peristalsis. In addition, the deposited Lewy bodies and *α*Syn reach the dorsal vagal nucleus through the vagus nerve and eventually enter and deposit in the substantia nigra and striatum [63], causing motor symptoms, NMSs, and constipation. Moreover, the importance of intestinal microbiota should not be ignored. Patients with PD have intestinal microbiota disturbance, and intestinal microbiota-derived metabolites stimulate the intestinal wall, disrupt the intestinal mucosal barrier, and enhance intestinal permeability, leading to constipation [64]. In the present study, we showed that electroacupuncture significantly increased the fecal output and accelerated the small-intestine propulsion of Thy1-*α*Syn mice, thereby improving the intestinal motility disorder. This result was consistent with the findings from clinical research. In addition, electroacupuncture upregulated 5-HT_4R_ expression in the mouse colon tissue, activated the downstream cAMP/PKA signaling pathway, and regulated the SP and CGRP neurotransmitter levels.

It is known that 5-HT_4R_ is an important target for treating constipation in PD and is closely related to gastrointestinal motility. The 5-HT_4R_ agonist mosapride has been widely used for treating constipation in PD. The related neurotransmitter 5-HT is involved in the regulation of various functions of the gastrointestinal tract [65]. Enterochromaffin cells release 5-HT when the intestinal tract is exposed to stimulants or stress, and great quantities of 5-HT are released from the intestinal tract to abnormally activate 5-HT_4R_ located in the submucosa of the gastrointestinal tract, causing intestinal dysfunction. Subsequently, 5-HT_4R_ activates the downstream cAMP/PKA pathway, which is closely related to gastrointestinal diseases. Moreover, 5-HT_4R_ in the gastrointestinal tract activates adenosine cyclase, which subsequently promotes the production of cAMP, further activating PKA. The potassium channels are closed, and the cell membrane is depolarized with the activation of PKA, causing the opening of calcium channels. The influx of calcium ions and the high concentration of calcium ions in the cells lead to the secretion and release of other neurotransmitters in the gastrointestinal tract, such as SP and CGRP. Among these neurotransmitters, SP, which is distributed in the colonic myenteric plexus and promotes the contraction of gastrointestinal smooth muscle, is an excitatory transmitter in motor neurons. CGRP is a vasodilator that inhibits the movement of the gastrointestinal tract. Both SP and CGRP are involved in regulating the function of the gastrointestinal tract. Under the influence of neurotransmitters, gastrointestinal smooth muscle accelerates the contractile function, thereby accelerating intestinal motility [66]. However, activated PKA phosphorylates cAMP response element binding protein, and then the cAMP response element binds to activate cAMP response element binding protein to affect smooth muscle and alter the functional activity of the gastrointestinal tract.

We used ELISA, RT-qPCR, WB, and IHC to analyze the expression of 5-HT_4R_ and cAMP/PKA signaling-related molecules to evaluate the regulatory effects of electroacupuncture on these molecules. Our results indicated that in the colon tissue of Thy1-*α*Syn mice, the 5-HT concentration, and the expression levels of 5-HT_4R_, cAMP, PKA, and SP were reduced, whereas the expression of CGRP was increased. Electroacupuncture upregulated the mRNA and protein expression levels of 5-HT_4R_ and enhanced the 5-HT concentration in the colon tissue of Thy1-*α*Syn mice, which affected the downstream cAMP/PKA pathway by increasing the cAMP concentration and upregulating PKA. The protein and mRNA expression levels of SP were also increased, whereas CGRP levels were reduced. These results suggest that the key target of electroacupuncture for treating constipation in PD may be 5-HT_4R_ via the cAMP/PKA pathway.

Acupuncture is a traditional Chinese medicine therapy with an extensive history and has unique advantages in the treatment of PD. Similar to natural medicines, acupuncture has fewer side effects and is therefore more suitable for long-term treatment of PD than synthetic drugs [67]. Acupuncture is currently used as a complementary and alternative therapy for PD in many countries [68–71]. Studies have shown that acupuncture significantly improves motor symptoms and NMSs in PD [37–39, 72–74]. Acupuncturists can accurately control the frequency and parameters and quantify the intensity of the stimulation. Our research group has been engaged in PD research for many years [75] and has confirmed the clinical efficacy of acupuncture for treating PD and the related mechanisms. We also found that electroacupuncture significantly improved the overall conditions, motor symptoms, and quality of life of patients with PD [40, 76]. In addition, we previously observed that electroacupuncture improved constipation symptoms in patients with PD, possibly due to the improvement of peristalsis of intestinal smooth muscle. Electroacupuncture also has a regulatory effect on the intestinal microbiota. Indeed, a previous study showed that electroacupuncture improved motor dysfunction by regulating the intestinal microbiota diversity in a 1-methyl-4-phenyl-1, 2, 3, 6-tetrahydropyridine-induced PD mouse model [77]. Similarly, our previous study showed that acupuncture prevented the intestinal microbiota from affecting the nervous system of PD mice by regulating the intestinal microbiota and improving the diversity of intestinal microorganisms, thereby protecting substantia nigra neurons [78]. The Thy1-*α*Syn transgenic PD mouse model used in this study is a classic model of intestinal motility disorders in PD [79–81]. The study by Sampson et al. also confirmed that this mouse model has intestinal motility disorders [54]. Our previous research confirmed that Thy1-*α*Syn mice had intestinal motility disorders and showed that electroacupuncture downregulated the protein expression of *α*Syn in the mouse colon tissue and promoted the contraction of colon smooth muscle, thereby improving intestinal motility disorders.

This study has some limitations. Regarding the study design, we did not use 5-HT_4R_ antagonists for reverse verification, which limits the strength of the evidence. In addition, we mainly focused on studying the intestine without comprehensively exploring the relationship between electroacupuncture and the brain-gut axis in PD. Specific antagonists should be used in further research to elucidate the regulatory mechanisms of electroacupuncture. Moreover, how electroacupuncture affects the intestines and substantia nigra in PD, as well as their potential interactions, would be an interesting future research direction.

In conclusion, we showed that electroacupuncture improved fecal excretion, accelerated small-intestine propulsion, and improved the intestinal motility of Thy1-*α*Syn mice. Electroacupuncture may relieve constipation in PD by upregulating 5-HT and 5-HT_4R_ in the colon tissue, thereby activating the downstream cAMP/PKA pathway, regulating the expression of the related neurotransmitters SP and CGRP, and further regulating the enteric nervous system to improve intestinal motility disorders. Our results preliminarily reveal the mechanisms by which electroacupuncture relieves constipation in PD mice and provide a basis for the clinical application of electroacupuncture as a complementary and alternative therapy for constipation in PD. However, further studies focusing on the underlying molecular mechanisms are needed.

## Figures and Tables

**Table 1:** Primer sequences for the RT-qPCR in this study.

Primer names	Primer sequences (5′ to 3′)
Mouse 5-HT_4R _F-primer	AGTTCCAACGAGGGTTTCAGG
Mouse 5-HT_4R_ R-primer	CAGCAGGTTGCCCAAGATG
Mouse PKA F-primer	AGATCGTCCTGACCTTTGAGT
Mouse PKA R-primer	GGCAAAACCGAAGTCTGTCAC
Mouse CGRP F-primer	CAGTGCCTTTGAGGTCAATCT
Mouse CGRP R-primer	CCAGCAGGCGAACTTCTTCTT
Mouse SP F-primer	TTTCTCGTTTCCACTCAACTGTT
Mouse SP R-primer	GTCTTCGGGCGATTCTCTGC
Mouse GAPDH F-primer	AGGTCGGTGTGAACGGATTTG
Mouse GAPDH-primer	TGTAGACCATGTAGTTGAGGTCA

**Table 2:** General conditions of mice in different groups after the corresponding interventions.

Postintervention conditions	Normal group	PD model group	Electroacupuncture group	Western medicine group
Appearance	Good	Poor	Average	Average
Behavioral performance	No tremor or shaking	Involuntary tremor and limb shaking	Involuntary tremor and limb shaking	Involuntary tremor and limb shaking
Defecation	Normal	Reduced	Increased	Increased

Electroacupuncture and western medicine interventions increased defecation in PD mice.

## Data Availability

The research data used to support the findings of this study are included within the article.
